# Activated mesangial cells induce glomerular endothelial cells proliferation in rat anti‐Thy‐1 nephritis through VEGFA/VEGFR2 and Angpt2/Tie2 pathway

**DOI:** 10.1111/cpr.13055

**Published:** 2021-05-13

**Authors:** Yinghua Zhao, Bo Fu, Pu Chen, Qinggang Li, Qing Ouyang, Chuyue Zhang, Guangyan Cai, Lingling Wu, Xiangmei Chen

**Affiliations:** ^1^ Department of Nephrology First Medical Center of Chinese PLA General Hospital Nephrology Institute of the Chinese People's Liberation Army State Key Laboratory of Kidney Diseases National Clinical Research Center for Kidney Diseases Beijing Key Laboratory of Kidney Disease Research Beijing China; ^2^ Department of Nephrology The Second Hospital of Jilin University Changchun China

**Keywords:** Angpt2/Tie2 pathway, glomerular endothelial cells, mesangial cells, mesangial proliferative glomerulonephritis, VEGFA/VEGFR2 pathway

## Abstract

**Objectives:**

We aimed to investigate the underlying mechanism of endothelial cells (ECs) proliferation in anti‐Thy‐1 nephritis.

**Materials and methods:**

We established anti‐Thy‐1 nephritis and co‐culture system to explore the underlying mechanism of ECs proliferation in vivo and in vitro. EdU assay kit was used for measuring cell proliferation. Immunohistochemical staining and immunofluorescence staining were used to detect protein expression. ELISA was used to measure the concentration of protein in serum and medium. RT‐qPCR and Western blot were used to qualify the mRNA and protein expression. siRNA was used to knock down specific protein expression.

**Results:**

In anti‐Thy‐1 nephritis, ECs proliferation was associated with mesangial cells (MCs)‐derived vascular endothelial growth factor A (VEGFA) and ECs‐derived angiopoietin2 (Angpt2). In vitro *co‐culture system* activated MCs‐expressed VEGFA to promote vascular endothelial growth factor receptor2 (VEGFR2) activation, Angpt2 expression and ECs proliferation, but inhibit TEK tyrosine kinase (Tie2) phosphorylation. MCs‐derived VEGFA stimulated Angpt2 expression in ECs, which inhibited Tie2 phosphorylation and promoted ECs proliferation. And decline of Tie2 phosphorylation induced ECs proliferation. In anti‐Thy‐1 nephritis, promoting Tie2 phosphorylation could alleviate ECs proliferation.

**Conclusions:**

Our study showed that activated MCs promoted ECs proliferation through VEGFA/VEGFR2 and Angpt2/Tie2 pathway in experimental mesangial proliferative glomerulonephritis (MPGN) and in vitro co‐culture system. And enhancing Tie2 phosphorylation could alleviate ECs proliferation, which will provide a new idea for MPGN treatment.

## Graphical abstract

The underlying mechanism of glomerular ECs proliferation. Normally, Angpt2 was stored in the Weibel‐Palade bodies in ECs. When MCs were stimulated, they produced VEGFA which acted on the ECs surface receptor VEGFR2 to promote Angpt2 expression. Angpt2 acted on Tie2 through autocrine which inhibited Tie2 phosphorylation and caused ECs proliferation. ECs, endothelial cells. MCs, mesangial cells. VEGFA, vascular endothelial growth factor A. VEGFR2, vascular endothelial growth factor receptor2. Angpt2, angiopoietin2. Tie2, TEK tyrosine kinase.

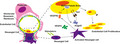

## 1 INTRODUCTION

Mesangial proliferative glomerulonephritis (MPGN) is characterized by mesangial cells (MCs) proliferation and increased extracellular matrix deposition.^1^, ^2^, ^3^, ^4^ As the most common pathological change of primary glomerulonephritis worldwide, MPGN leads to glomerular fibrosis and sclerosis and eventually results in end‐stage renal disease.^5^ Anti‐Thy‐1 nephritis is the most suitable animal model to study the specific pathogenesis of MPGN.^5^, ^6^ It shows that as MCs were damaged, glomerular capillaries proliferated and developed aneurysmal changes,^7^ and as MCs continued to return to normal, the “aneurysm” gradually decreased.^8^, ^9^ Therefore, there might be cell‐cell communication between MCs and ECs, which leads that mesangial areas and the capillaries change synchronously. And strategies to prevent such cell‐cell communication would be useful in the resolution of vascular lesions in MPGN.

Vascular endothelial growth factor (VEGF) plays significant role in angiogenesis, tumour growth, development and atherosclerosis, which mainly produced by podocytes, distal tubules and collecting duct epithelial cells in normal human and rat kidneys.^10^ As a dimer protein, it composed of 121, 165, 189 or 206 amino acids, respectively. VEGF_165_, VEGFA, is the most common isoform and it has been verified that VEGFA expressed in activated MCs of experimental MPGN.^11^ TEK tyrosine kinase (Tie2) is expressed on the surface of ECs and is a tyrosine kinase receptor with two homologous domains, IG and EGF.^12^ Angiopoietin1 (Angpt1) and angiopoietin2 (Angpt2) competitively bind to Tie2, which is important in angiogenesis and vascular inflammation. Angpt1 binds to Tie2 to promote Tie2 phosphorylation, thereby maintaining vascular structural integrity, protecting ECs from apoptosis and inhibiting the inflammatory response.^13^, ^14^, ^15^ Angpt2 is expressed and stored in the Weibel‐Palade body of ECs, interfering with the Angpt1/Tie2 signalling pathway.^16^, ^17^ VEGFA and Angpt2 both are considered as pro‐angiogenesis factors.^18^, ^19^, ^20^ Martina Schmittnaegel et al pointed out that Angpt2 and VEGFA bispecial‐antibody (A2V) has a better therapeutic effect than single‐antibody on metastatic breast cancer, pancreatic neuroendocrine tumour and melanoma.^21^ And the phase 2 randomized trial of Faricimab (also a novel bispecific antibody targeting Angpt2 and VEGFA) showed that it could benefit diabetic macular oedema.^22^ Even though many studies indicated that VEGFA and Angpt2 had synthetic effects in angiogenesis,^21^, ^23^ there are few observations of their roles in glomerular angiogenesis. Therefore, we tried to investigate whether VEGFA and Angpt2 participated in glomerular vascular lesions.

In this study, the rat anti‐Thy‐1 nephritis model and cell co‐culture system were used to explore the specific mechanism of vascular lesions, and we try to alleviate ECs proliferation by blocking the cell‐cell communication so as to provide new ideas for disease treatment.

## 2 MATERIALS AND METHODS

### 2.1 Animals and biochemical measurements

Wild‐type Wistar rats (male, aged 6‐8 weeks, 200‐250 g) were purchased from the Animal Center of PLA General Hospital. All rats were kept in an environment with constant temperature (20°C), humidity (70%) and alternating day and night cycles. To explore the glomerular endothelial cells (ECs) proliferation in anti‐Thy‐1 nephritis, the rats were divided into control group (n = 7) and model group (n = 7). Anti‐Thy‐1 antibody (2.5 mg/kg) was administered by tail vein injection to sober rats in the model group to establish rat anti‐Thy‐1 nephritis. The control group was injected with saline solution. To evaluate the role of Vasculotide in ECs proliferation, we divided the rats into a control group (n = 7), model group (n = 7) and Vasculotide group (n = 7). The Vasculotide group was repeatedly injected with Vasculotide (3 µg/kg, ip Method and dose of administration reference before^24^) on the 4th, 5th and 6th days, and model group injected with phosphate buffer saline (PBS). On the 7th day, the rats were sacrificed after sedated by pentobarbital. Glomeruli were isolated from kidney samples as described previously.^25^ The experiments were performed according to the Guide for the Care and Use of Laboratory Animals (National Research Council of the USA, 1996).

The urine obtained was centrifugated (900*g* for 30 minutes at 22°C) and stored at −80°C until used. To evaluate the enzymatic method for assaying creatinine in urine by using Creatinine Assay Kit (C011‐2‐1, Nanjing Jiancheng Bioengineering Institute), urine albumin was measured by CBB method using Urine Protein Test Kit (C035‐2‐1, Nanjing Jiancheng Bioengineering Institute). Urine albumin creatinine ratio (UACR, mg/mmol) was calculated by urine albumin/creatinine.

The blood was collected in vacutainer tubes and centrifugated (100g for 15 minutes at 22°C). Then, the upper serum was left and stored at −80°C until used. Angpt2 in serum was measured by using Angiopoietin‐2 Quantikine ELISA Kit (R&D Systems) according to manufacturer's instructions.

### 2.2 Vasculotide

Tournaire et al^26^ were the first to synthesize a short peptide HHHRHSF whose tetramer forming by affinity/biotin could combine with the extracellular part of Tie2 to induce Tie2 phosphorylation. At present, 4‐arm polyethanol glycol (average molecular weight 10 kDa) scaffold is used to replace the avidin/biotin complex. In our study, Vasculotide was synthesized by Shanghai Bootech Bioscience & Technology Co (Figure S7).

### 2.3 Renal tissue staining

#### 2.3.1 Periodic Acid‐Schiff (PAS) staining

Rat kidneys were fixed in 10% formalin and dehydrated with gradient ethanol. The tissue was embedded in paraffin and sectioned into 2‐4 µm slices. Then, the tissue slices were stained with PAS. The mesangial hypercellularity index was used to assess the severity of hypercellular lesions as follows: 0, no hypercellularity, with fewer than three cells per mesangial area; 1, mild focal hypercellularity and <50% of glomeruli with three to five cells per mesangial area; 2, diffuse mild hypercellularity or prominent focal segmental hypercellularity with more than five cells per mesangial area; and 3, prominent, diffuse global hypercellularity. Twenty glomeruli were selected for each section.

#### 2.3.2 Immunohistochemical staining

Paraffin sections (2‐4 µm) were dewaxed with xylene and ethanol, incubated with 3% hydrogen peroxide and heated in a microwave for antigen retrieval. The sections were blocked with normal goat serum (ZLI‐9056, ORIGENE, CN) and incubated with rat endothelial cells antigen‐1 (RECA‐1) antibody (1:100, Abcam, cat. ab22492) or proliferative cells nuclear antigen (PCNA) antibody (1:10 000, Abcam, cat. ab92552) overnight at 4°C. The indirect avidin‐biotinylated peroxidase complex method (Vecta‐Stain Elite ABC Kit, Vector Laboratories) with secondary antibody was used. PBS was used instead of primary antibodies as a negative control. The PCNA‐positive rates are represented as the ratio of positive cells per glomerulus. ImageJ was used for RECA‐1 analysis. Twenty glomeruli were counted in each section.

#### 2.3.3 Immunofluorescence staining

Frozen sections were sequentially treated with 1% SDS and normal goat serum before being incubated with RECA‐1 (1:200, Abcam, cat. ab22492)/PCNA antibody (1:200, Abcam, cat. ab92552), RECA‐1/Angpt2 (1:200, Abcam, cat. ab8452) antibody, Thy‐1/Angpt2 antibody, VEGFA (1:100, Abcam, cat. ab214424)/Thy‐1 antibody, or VEGFA/Wilms tumour protein1 (WT‐1, 1:100, Abcam, ab220212) overnight at 4°C. The sections were washed and probed with Cy3‐conjugated secondary antibody (red) and FITC‐conjugated secondary antibody (green) at room temperature for 1 hour. DAPI was added to stain the nuclei. The tissue sections were imaged by confocal fluorescence microscopy. Each experiment was repeated three times.

### 2.4 Cell culture

Primary human renal mesangial cells (HRMCs) were purchased from ScienCell Research Laboratories (cat. 4200) and cultured in mesangial cell medium (MCM, cat. 4201) supplemented with 10% FBS (cat. 0010), 5 mL of mesangial cell growth factor (MsCGS, cat. 4252) and 5 mL of penicillin/streptomycin (P/S) solution (cat. 0503). Primary human glomerular endothelial cells (HRGECs) were purchased from ScienCell Research Laboratories (cat. 4000) and cultured in endothelial cell medium (ECM, cat. 1001) supplemented with 10% FBS, 5 mL of endothelial cell growth factor (ECGS, cat. 1052) and 5 mL of P/S solution. Cells were all maintained in 5% CO_2_ at 37°C. PDGF‐BB (Peprotech, cat. 100‐14b, 20 ng/mL) and VEGFA (Peprotech, cat. 100‐20, 50 ng/mL) were used as irritants of MCs and ECs, respectively.

### 2.5 Cell co‐culture

Transwells were purchased from Corning and used for co‐culturing. MCs were seeded in transwell inserts and were cultured alone or stimulated with platelet‐derived growth factor BB (PDGF‐BB, to activate MCs) treatment for 24 hours and were then washed with fresh medium followed by co‐culturing with ECs for another 24 hours. During co‐culturing, anti‐VEGFA neutralizing antibody (R&D, cat. ab‐293‐na, 3 µg/mL) or anti‐Angpt2 neutralizing antibody (Abcam, cat. Ab155106, 2 µg/mL) was added to the co‐culture system to block VEGFA or Angpt2, and recombinant human Angpt1 (Prospec, cat. Cyt‐074, 0.7 µg/mL) and Angpt2 (MCE, cat. HY‐P7510, 0.5 µg/mL) was used to influence Tie2 phosphorylation.

### 2.6 VEGFA/VEGFR2 and Angpt2/Tie2 signalling pathway analysis

#### 2.6.1 Cell proliferation analysis

Cell proliferation was analysed by the Click‐iT^®^ Plus EdU Alexa Fluor^®^ 555 Imaging Kit (Invitrogen, cat. C10638) according to the manufacturer's instructions. The cells were co‐cultured in transwell plates, and 10 µmol/L EdU was added to the medium for 24 hours. Then, the cells were fixed with 4% paraformaldehyde, permeabilized with 0.5% Triton X‐100 and washed with 3% BSA. The cells were then incubated at room temperature in the Click‐iT^®^ reaction cocktail for 30 minutes. DAPI was used to dye the nuclei. Cells were observed under a fluorescence microscope (Olympus). All experiments were repeated three times.

#### 2.6.2 Immunofluorescence staining

Vascular endothelial growth factor A (1:100, Abcam, cat. ab214424) was stained with desmin (1:100, Santa Cruz, sc‐23879) to evaluate the VEGFA expression in MCs. Angpt2 (1:200, Abcam, cat. ab221154) or p‐tie2 (1:100, Biorbyt Ltd., orb7094) was stained with CD31 (1:500, Abcam, cat. ab24590) to evaluate changes in Angpt2 expression and Tie2 phosphorylation in ECs.

#### 2.6.3 Real‐time quantitative PCR (RT‐qPCR)

TRIzol reagent (Invitrogen) was used to lyse the cells and extract the RNA. The ReverTra Ace qPCR RT kit (Toyobo) reverse‐transcribed the RNA into cDNA. RT‐qPCR was performed using SYBR Select master mix (Life Technologies) and an RT‐qPCR detection system (ABI). The primers for the following genes were constructed: VEGFA‐homo (5′‐agggcagaatcatcacgaagt‐3′; 5′‐agggtctcgattggatggca‐3′), Angpt2‐homo (5′‐accccactgttgctaagaaga‐3′; 5′‐ccatcctcacgtcgctgaata‐3′), VEGFA‐rattus (5′‐ gggagcagaaagcccatgaa‐3′; 5′‐gctggctttggtgaggtttg‐3′), Angpt2‐rattus (5′‐ tccagactgacgcacatcac‐3′; 5′‐atttctccagacccgcagtg‐3′). The 18s gene was used as an internal control, and the expression of each target gene was calculated by the 2^−ΔΔCT^ method. All experiments were repeated three times.

#### 2.6.4 Western blot

Glomeruli from renal tissue or cells were lysed with RIPA lysis buffer containing protease inhibitors (1 µg/mL leupeptin, 1 µg/mL aprotinin and 100 µmol/L PMSF). After a 30 minutes incubation, the samples were centrifuged at 13800 *g* and 4°C for 30 minutes. The protein concentration was determined by a BCA protein assay kit (Thermo Fisher Scientific). Approximately 40 µg of protein from each sample was separated by 8%‐15% SDS‐PAGE. The samples were transferred from the SDS‐PAGE gels to membranes. The membranes were blocked and incubated in antibodies against CD34 (protein marker of ECs, 1:1000, Abcam, ab81289), VEGFA (1:1000, Abcam, cat. ab214424), Angpt2 (1:500, Abcam, cat. ab221154), Tie2 (1:1000, Biorbyt Ltd., orb247862), p‐tie2 (1:1000, Biorbyt Ltd., orb7094), vascular endothelial growth factor receptor 2 (VEGFR2, 1:1000, CST, 2479s), p‐VEGFR2 (1:1000, CST, 2478s) and β‐actin overnight at 4°C. Finally, the membranes were incubated with secondary antibody at room temperature for 2 hours. ImageJ was used for blot analysis. All experiments were repeated three times.

#### 2.6.5 ELISA

The ECs medium was collected after ECs co‐cultured with MCs and centrifuged (100 *g* for 15 minutes at 4°C). Then, the supernatant was left and stored at −80°C until used. Angpt2 in medium was measured by using Angiopoietin‐2 Quantikine ELISA Kit (R&D Systems) according to manufacturer's instructions.

#### 2.6.6 Small interfering (si) RNA transfection

Endothelial cells were spread onto 1.7 cm^2^ culture dishes (Millicell EZ Slide). When cells reached 70%‐80% confluence, the cells were transfected with 50 nmol/L Tie2 siRNA, Angpt2 siRNA, or negative control using siRNA Transfection Reagent (Shanghai GenePharma CO., Ltd.) according to manufacturer's instructions. After 8 hours of transfection, cells were exposed to stimulating factors.

### 2.7 Statistical analysis

All data analyses were performed using GraphPad (version 5.0; GraphPad). The data are expressed as the means ± SD. Comparisons among the groups were conducted using Student *t* test. A value of *P* < .05 was considered significant.

## 3 RESULTS

### 3.1 On the 7th day of anti‐Thy‐1 nephritis, ECs proliferated and the expressions of VEGFA and Angpt2 increased

**FIGURE 1:**
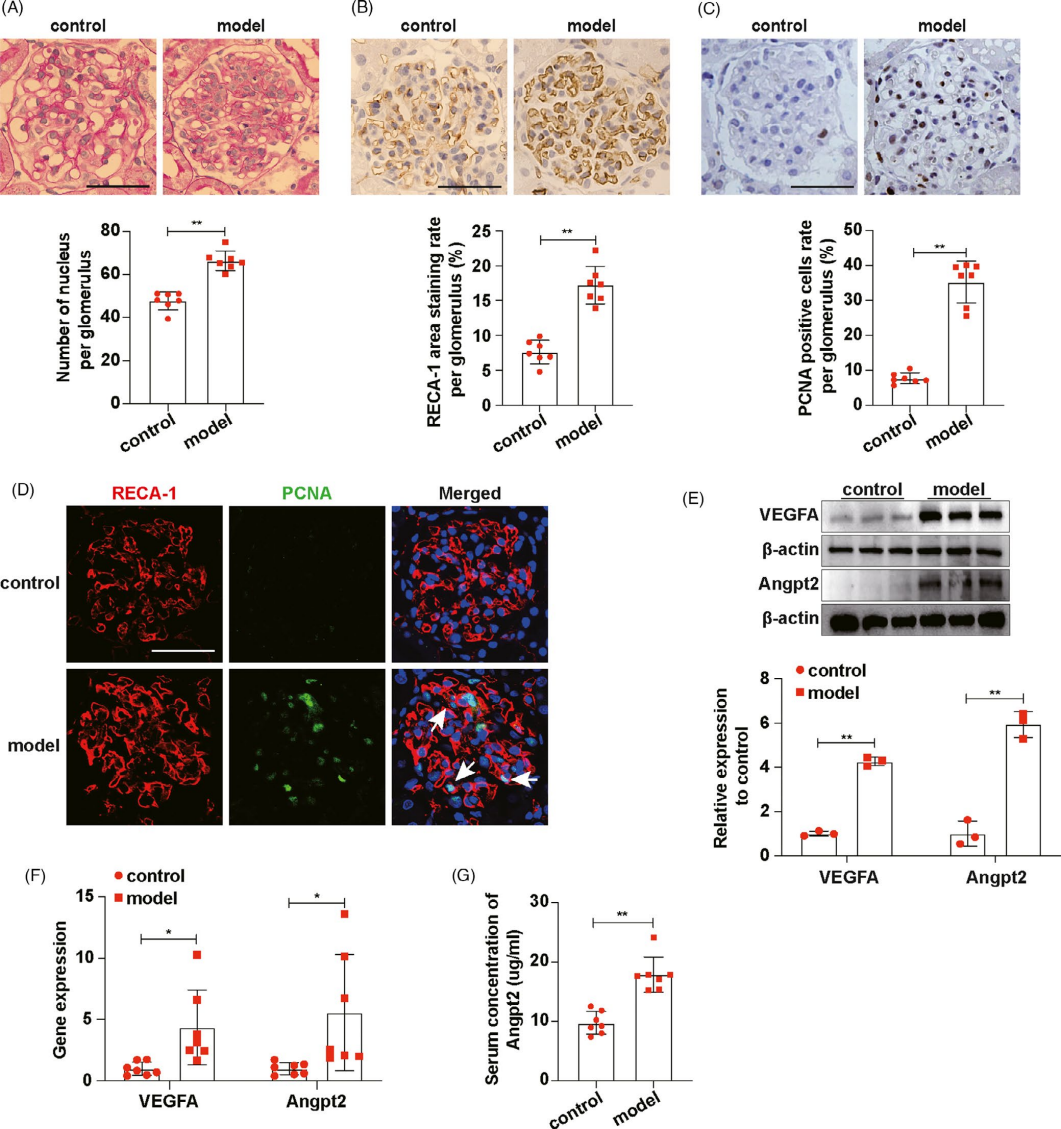
On the 7th day of anti‐Thy‐1 nephritis, ECs proliferated and the expressions of VEGFA and Angpt2 increased. A, PAS staining showed glomerulopathy, and the number of nucleus per glomerulus was qualified (n = 7). B, RECA‐1 expressions in glomeruli were detected by immunohistochemical staining and RECA‐1 area staining rate per glomerulus was qualified (n = 7). C, PCNA expressions in glomeruli were detected by immunohistochemical staining and PCNA‐positive cells rate per glomerulus was qualified (n = 7). D, Locations of RECA‐1 and PCNA expressions were showed by immunofluorescence co‐staining. White arrows indicated proliferating ECs. RECA‐1, red. PCNA, green. DAPI, blue. E, VEGFA and Angpt2 expressions in glomeruli were detected by Western blot. F, mRNA expression levels of VEGFA and Angpt2 in glomeruli were detected by RT‐qPCR (n = 7). G, Concentrations of Angpt2 in rat serum were determined by ELISA (n = 7). Scale bars, 50 μm. Results are presented as the mean values (±SD), *P < .05, **P < .01. Angpt2, angiopoietin2; ECs, endothelial cells; PCNA, proliferative cells nuclear antigen; RECA‐1, rat endothelial cells antigen‐1; VEGFA, vascular endothelial growth factor A. Control, normal rats. Model, anti‐Thy‐1 nephritis rats

The rat anti‐Thy‐1 nephritis model was successfully established, according to increased urine albumin/creatinine ratio (UACR) in model group (Figure S1A). PAS staining results showed that the number of cells in the glomeruli increased significantly on the 7th day after the model was established (Figure 1A). Moreover, rat endothelial cells antigen‐1 (RECA‐1) and proliferative cells nuclear antigen (PCNA) immunohistochemical staining (Figure 1B,C) and immunofluorescence co‐staining (Figure 1D) results showed that the number of glomerular endothelial cells (ECs) increased. Western blot results showed that expression of CD34 (protein marker of ECs) was also increased significantly in the model group (Figure S1B). These results indicated that there was abnormal ECs proliferation on the 7th day after the model was established. Western blot and RT‐qPCR results showed that the expressions of vascular endothelial growth factor A (VEGFA) and angiopoietin2 (Angpt2) in glomeruli of the model group were increased (Figure 1E,F). Moreover, serum Angpt2 levels of model group were higher than control (Figure 1G). VEGFA/Thy‐1 (protein marker of MCs) and VEGFA/Wilms tumour protein‐1 (WT‐1, protein marker of podocyte) immunofluorescence co‐staining results showed that the expression of VEGFA apparently increased in the mesangial area of glomerular in the model group (Figure S1C,D). Angpt2/RECA‐1 (protein marker of ECs) and Angpt2/Thy‐1 immunofluorescence co‐staining results showed that most of Angpt2 was expressed by ECs (Figure S1E,F). Therefore, we suspected that MCs‐derived VEGFA and ECs‐derived Angpt2 might play important roles in ECs proliferation of anti‐Thy‐1 nephritis.

### 3.2 MCs promoted ECs proliferation through VEGFA/VEGFR2 pathway

**FIGURE 2:**
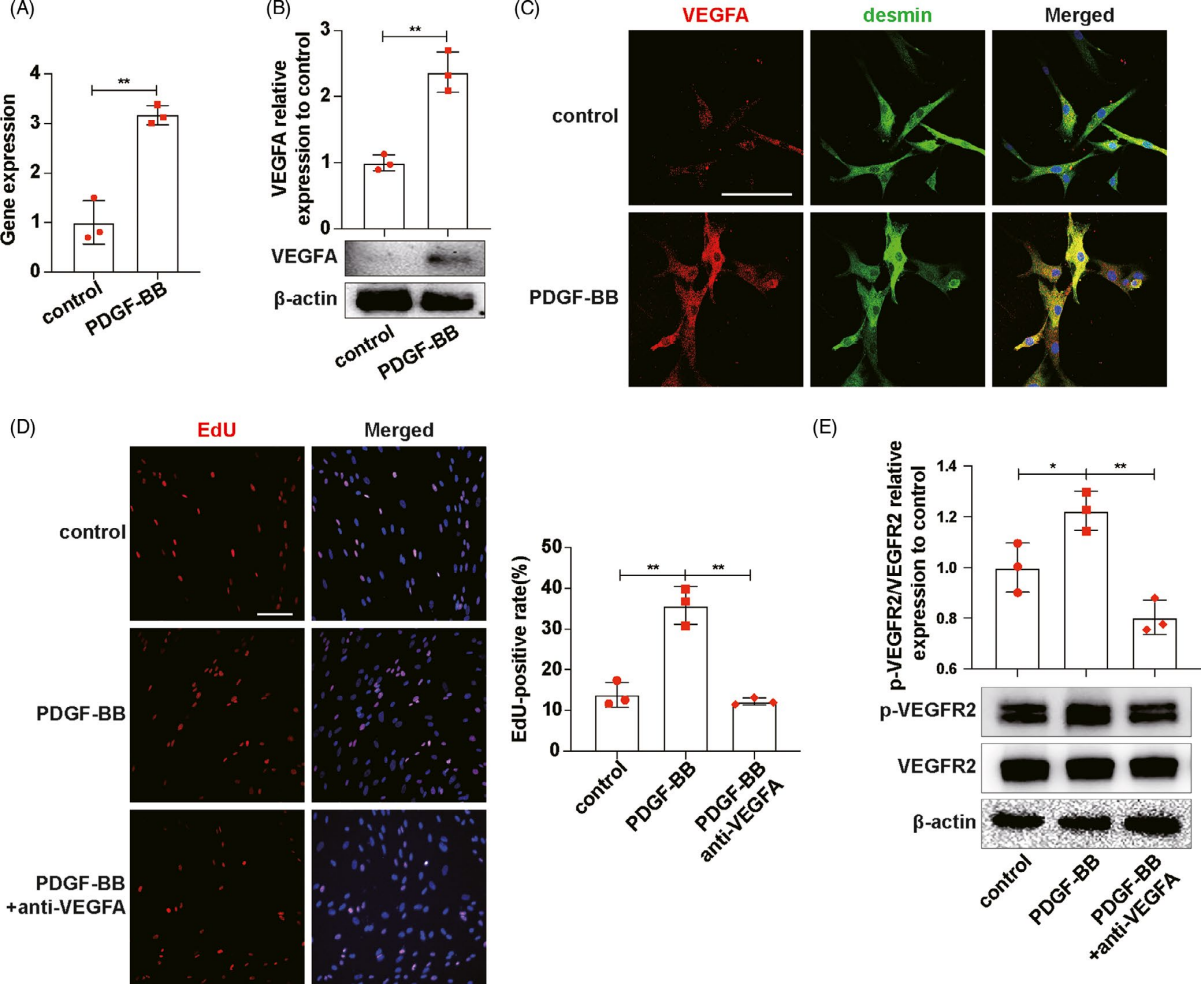
MCs promoted ECs proliferation through VEGFA/VEGFR2. A, mRNA expression levels of VEGFA in MCs were detected by RT‐qPCR. B, VEGFA expressions in MCs were detected by Western blot. C, VEGFA expressions in MCs were detected by VEGFA/desmin immunofluorescence co‐staining. VEGFA, red. desmin, green. DAPI, blue. D, EdU was used to detect ECs proliferation. E, p‐VEGFR2 and VEGFR2 expressions on ECs were detected by Western blot. Results are presented as the mean values (±SD), *P < .05, **P < .01. Scale bars, 50 μm. Desmin, protein marker of MCs; ECs, endothelial cells; MCs, mesangial cells; PDGF‐BB, platelet‐derived growth factor BB, to activate MCs; VEGFA, vascular endothelial growth factor A; VEGFR2, vascular endothelial factor receptor2. (A, B, C) Control, inactive MCs. PDGF‐BB, MCs stimulated by PDGF‐BB for 24 h. (D, E) Control, ECs co‐cultured with inactive MCs. PDGF‐BB, ECs co‐cultured with PDGF‐BB‐activated MCs. PDGF‐BB+anti‐VEGFA, ECs co‐cultured with PDGF‐BB‐activated MCs and VEGFA neutralizing antibody, was added

Mesangial cells were seeded in transwell inserts and were cultured alone or stimulated with platelet‐derived growth factor BB (PDGF‐BB, to activate MCs) treatment for 24 hours and were then washed with fresh medium followed by co‐culturing with ECs for another 24 hours (Figure S2A). The results showed that activated MCs promoted ECs proliferation (Figure S2B), which indicated activated MCs could impact ECs. The RT‐qPCR, Western blot and VEGFA/desmin (protein marker of MCs) immunofluorescence co‐staining results showed that the expression of VEGFA in PDGF‐BB‐activated MCs was significantly increased (Figure 2A‐C). Then, to investigate whether ECs proliferation was stimulated by MCs‐derived VEGFA, VEGFA neutralizing antibody was added in the co‐culture system (Figure S2C). EdU assay results showed that adding VEGFA neutralizing antibody in co‐culture system could inhibit ECs proliferation (Figure 2D). Western blot pointed that activated MCs can definitely activate vascular endothelial growth factor receptor 2 (VEGFR2), which was inhibited by VEGFA neutralizing antibody (Figure 2E). Thus, by demonstrating that blocking VEGFA in co‐culture system could inhibit ECs proliferation and VEGFR2 activation, we concluded that MCs generated VEGFA to activate VEGFR2 and promote ECs proliferation.

### 3.3 MCs‐derived VEGFA stimulated Angpt2 expression to promote ECs proliferation

**FIGURE 3:**
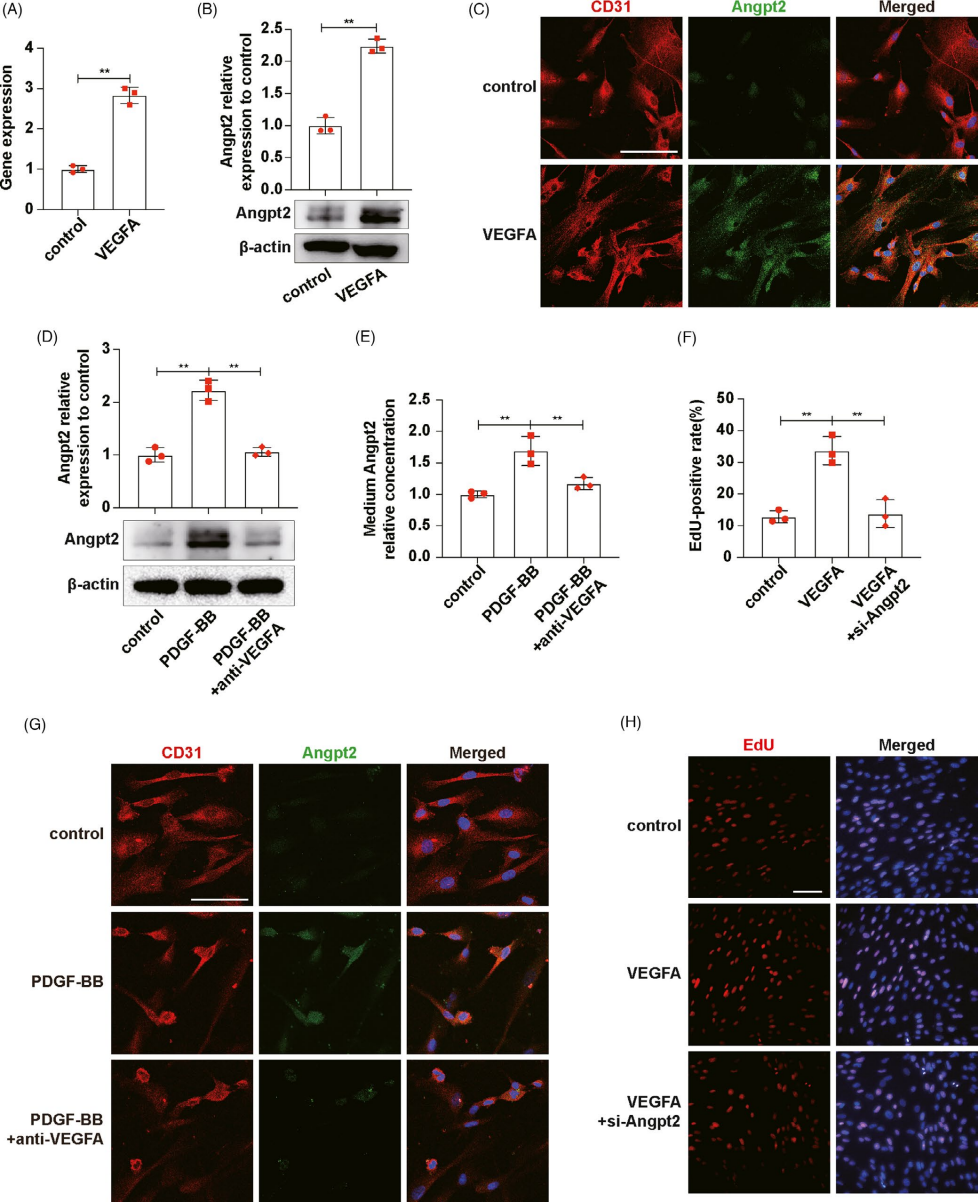
MCs‐derived VEGFA stimulated Angpt2 expression to promote ECs proliferation. A, mRNA expression levels of Angpt2 in ECs were detected by RT‐qPCR. B, Angpt2 expressions in ECs were detected by Western blot. C, Angpt2 expressions in MCs were detected by Angpt2/CD31 immunofluorescence co‐staining. CD31, red. Angpt2, green. DAPI, blue. D, Angpt2 expressions in ECs were detected by Western blot. E, Medium Angpt2 relative concentrations were measured by ELISA. F, Qualifications of EdU‐positive rates in H. G, Angpt2 expressions in ECs were detected by Angpt2/CD31 immunofluorescence co‐staining. CD31, red. Angpt2, green. DAPI, blue. H, EdU was used to detect ECs proliferation. Results are presented as the mean values (±SD), *P < .05, **P < .01. Scale bars, 50 μm. Angpt2, angiopoietin2; CD31, protein marker of ECs; ECs, endothelial cells; MCs, mesangial cells; PDGF‐BB, platelet‐derived growth factor BB, to activate MCs; VEGFA, vascular endothelial growth factor A. (A, B, C) Control, inactive ECs. VEGFA, ECs stimulated by VEGFA for 24 h. (D, E, G) Control, ECs co‐cultured with inactive MCs. PDGF‐BB, ECs co‐cultured with PDGF‐BB‐activated MCs. PDGF‐BB+anti‐VEGFA, ECs co‐cultured with PDGF‐BB‐activated MCs and VEGFA neutralizing antibody, was added. (F, H) control, inactive ECs. VEGFA, ECs stimulated by VEGFA for 24 h. VEGFA+si‐Angpt2, ECs stimulated by VEGFA for 24 h after transfected with si‐Angpt2

The RT‐qPCR, Western blot and Angpt2/CD31 (protein marker of ECs) immunofluorescence co‐staining results showed that VEGFA enhanced Angpt2 expression in ECs (Figure 3A‐C). Then, we co‐cultured ECs with activated MCs and added VEGFA neutralizing antibody in the co‐culture system. Western blot and immunofluorescence staining results showed that activated MCs promoted ECs to express Angpt2, which could be abrogated by VEGFA neutralizing antibody (Figure 3D,G). Moreover, we measured the concentration of Angpt2 in ECs medium by ELISA. The results showed that after co‐culturing activated MCs and ECs, the concentration of Angpt2 in the medium increased, while the concentration of Angpt2 decreased by adding VEGFA neutralizing antibody, which was consistent with the expression of Angpt2 in ECs (Figure 3E). Subsequently, we further explored the effect of Angpt2 on ECs proliferation. Transduction of Angpt2 siRNA in ECs significantly reduced the expression of Angpt2 protein (Figure S3). EdU results indicated that VEGFA could improve ECs proliferation, and this improvement could be diminished by knockdown of Angpt2 (Figure 3F,H). It can be seen that VEGFA expressed by MCs stimulated Angpt2 expression of ECs which promoted ECs proliferation.

### 3.4 MCs‐derived VEGFA stimulated Angpt2 expression which inhibited Tie2 phosphorylation on ECs

**FIGURE 4:**
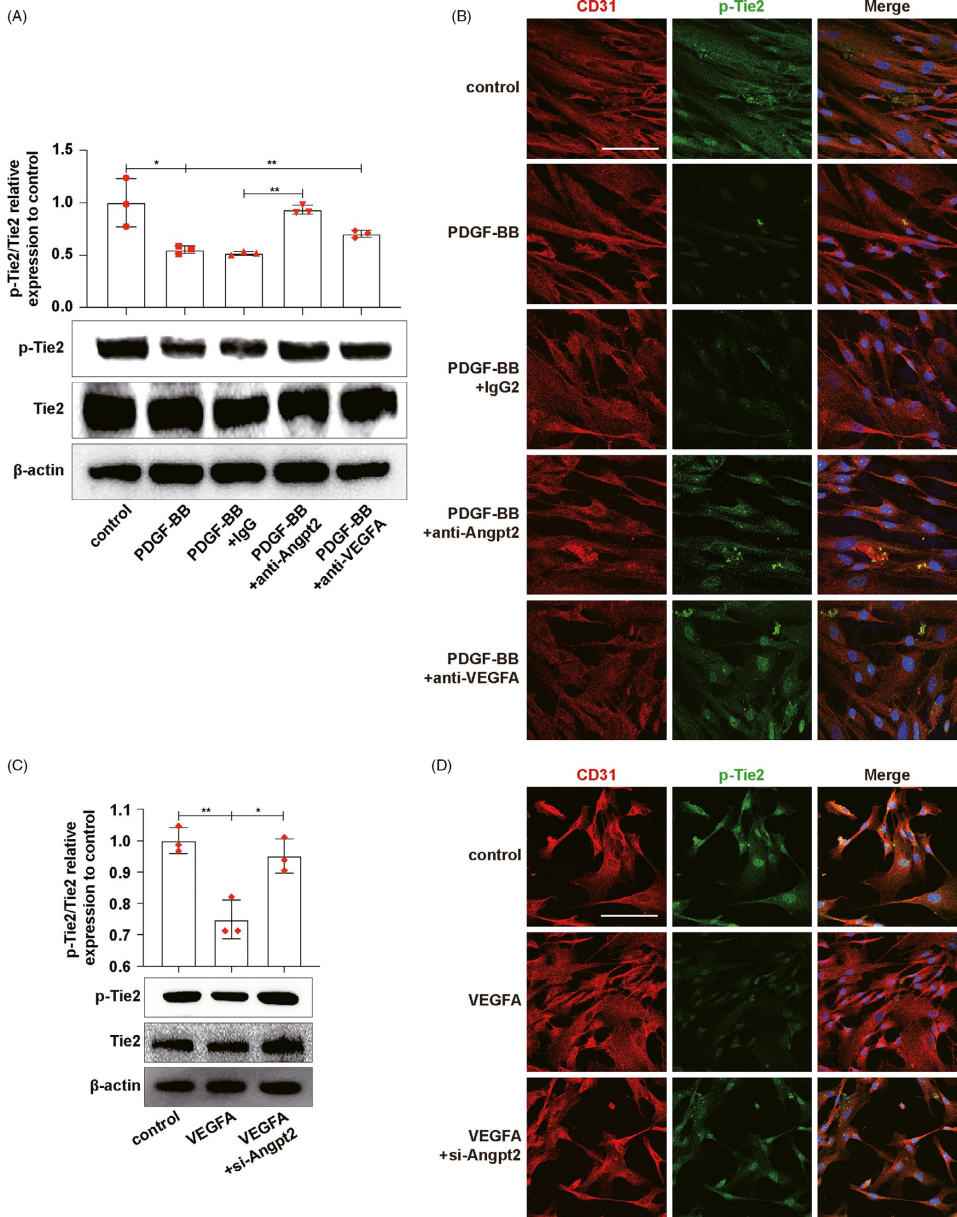
MCs‐derived VEGFA stimulated Angpt2 expression which inhibited Tie2 phosphorylation on ECs. A, C, p‐Tie2 and Tie2 expressions on ECs were detected by Western blot. B, D, p‐Tie2 expressions on ECs were detected by p‐Tie2/CD31 immunofluorescence co‐staining. CD31, red. p‐Tie2, green. DAPI, blue. Results are presented as the mean values (±SD), *P < .05, **P < .01. Scale bars, 50 μm. Angpt2, angiopoietin2; ECs, endothelial cells; MCs, mesangial cells; PDGF‐BB, platelet‐derived growth factor BB, to activate MCs; Tie2, TEK tyrosine kinase. (A, B) Control, ECs co‐cultured with inactive MCs. PDGF‐BB, ECs co‐cultured with PDGF‐BB‐activated MCs. PDGF‐BB+IgG, ECs co‐cultured with PDGF‐BB‐activated MCs and IgG antibody, was added as negative control. PDGF‐BB+anti‐Angpt2, ECs co‐cultured with PDGF‐BB‐activated MCs and Angpt2 neutralizing antibody, was added. PDGF‐BB+anti‐VEGFA, ECs co‐cultured with PDGF‐BB‐activated MCs and VEGFA neutralizing antibody, was added. (C, D) Control, inactive ECs. VEGFA, ECs stimulated by VEGFA for 24 h. VEGFA+si‐Angpt2, ECs stimulated by VEGFA for 24 h after transfected with si‐Angpt2

We continued to co‐culture MCs and ECs and add VEGFA neutralizing antibody or Angpt2 neutralizing antibody into the co‐culture system to investigate the effects of VEGFA and Angpt2 on Tie2 phosphorylation (Figure S4). The results of Western blot and p‐Tie2/CD31 immunofluorescence co‐staining showed that blocking VEGFA or Angpt2 could increase the phosphorylation levels of Tie2 (Figure 4A,B). So, VEGFA and Angpt2 inhibited Tie2 phosphorylation on ECs. Then, we knocked down Angpt2 in ECs by transfecting Angpt2 siRNA. Western blot and p‐Tie2/CD31 immunofluorescence co‐staining results present that the Tie2 phosphorylation levels of ECs decreased after exposing to VEGFA, while rose again after knocking down Angpt2 (Figure 4C,D), which indicated that VEGFA inhibited Tie2 phosphorylation through Angpt2. Based on the above results, we concluded that VEGFA derived from activated MCs could inhibit the phosphorylation of Tie2 by promoting Angpt2 expression in ECs.

### 3.5 Enhancing Tie2 phosphorylation can effectively alleviate activated MCs‐induced ECs proliferation

**FIGURE 5:**
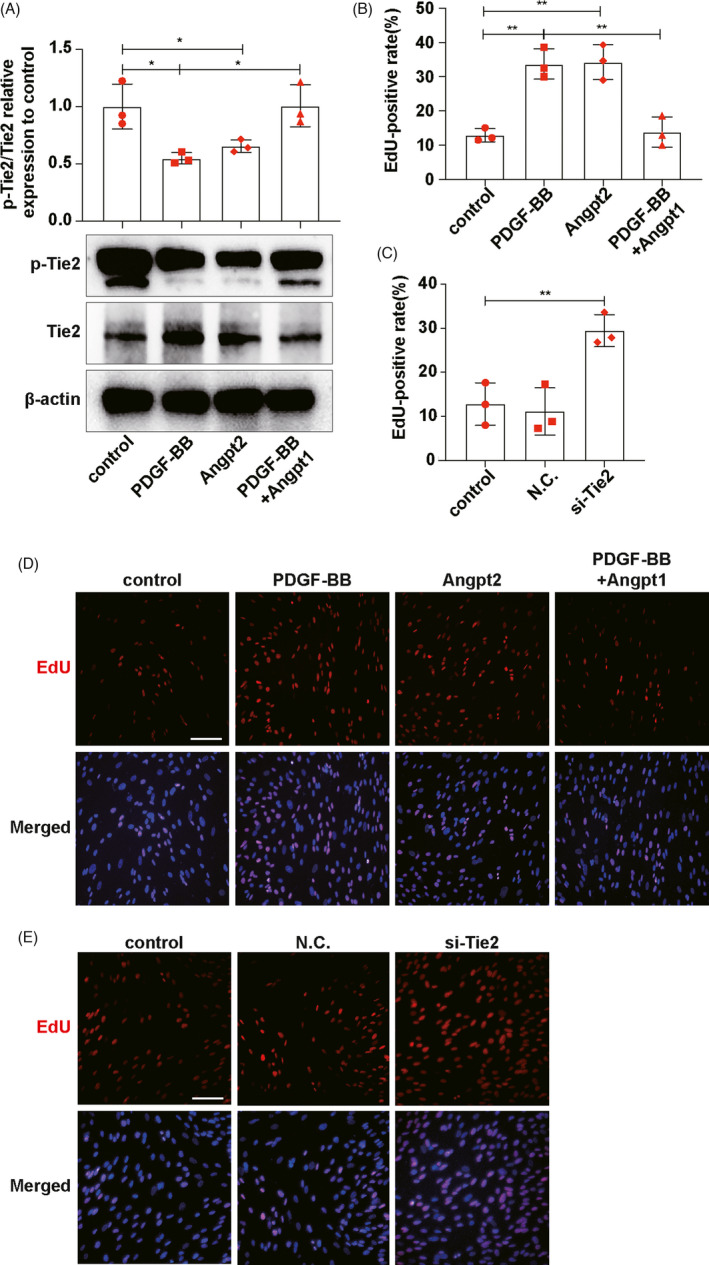
Enhancing Tie2 phosphorylation can effectively alleviate activated MCs‐induced ECs proliferation. A, p‐Tie2 and Tie2 expressions on ECs were detected by Western blot. B, Qualifications of EdU‐positive rates in D. C, Qualifications of EdU‐positive rates in E. D, E, EdU was used to detect ECs proliferation. Results are presented as the mean values (±SD), *P < .05, **P < .01. Scale bars, 50 μm. Tie2, TEK tyrosine kinase. MCs, mesangial cells. ECs, endothelial cells. Angpt2, angiopoietin2. Angpt1, angiopoietin. PDGF‐BB, platelet‐derived growth factor BB, to activate MCs. (A, B, D) Control, ECs co‐cultured with inactive MCs. PDGF‐BB, ECs co‐cultured with PDGF‐BB‐activated MCs. Angpt2, ECs co‐cultured with inactive MCs and Angpt2, was added. PDGF‐BB+Angpt1, ECs co‐cultured with PDGF‐BB‐activated MCs and Angpt1, was added. (C, E) Control, normal ECs. NC, ECs transfected with negative control siRNA. si‐Tie2, ECs transfected with si‐Tie2

Angiopoietin1 (Angpt1) and Angpt2 can bind to the same part of Tie2, but their effects on Tie2 phosphorylation are opposite. Angpt1 promotes Tie2 phosphorylation, which is inhibited by Angpt2. We added Angpt2 or Angpt1 in co‐culture system and detected the changes of Tie2 phosphorylation and ECs proliferation under different circumstances, so as to explore the relationship between Tie2 phosphorylation and ECs proliferation (Figure S5A). The results showed that activated MCs inhibited the phosphorylation of Tie2 on ECs, but Tie2 phosphorylation increased after adding Angpt1 to co‐culture system (Figure 5A). At the same time, Tie2 phosphorylation decreased after the addition of Angpt2 into the co‐culture system of static MCs and ECs (Figure 5A). EdU results showed that activated MCs enhanced ECs proliferation, but ECs proliferation was inhibited after adding Angpt1 to co‐culture system (Figure 5B,D). Moreover, ECs proliferation was boosted after the addition of Angpt2 into the co‐culture system of static MCs and ECs (Figure 5B,D). To further confirm the relationship between ECs proliferation and Tie2, we transfected si‐Tie2 to knock down Tie2 expression (inhibited Tie2 phosphorylation) in ECs (Figure S5B). And EdU results showed that the proliferation ability of ECs was significantly improved by knocking down Tie2 (Figure 5C,E). In conclusion, the proliferation ability of ECs was correlated with Tie2 phosphorylation levels, and the proliferation ability of ECs increased when Tie2 phosphorylation levels decreased.

### 3.6 Enhancing Tie2 phosphorylation can effectively alleviate ECs proliferation in anti‐Thy‐1 nephritis

**FIGURE 6:**
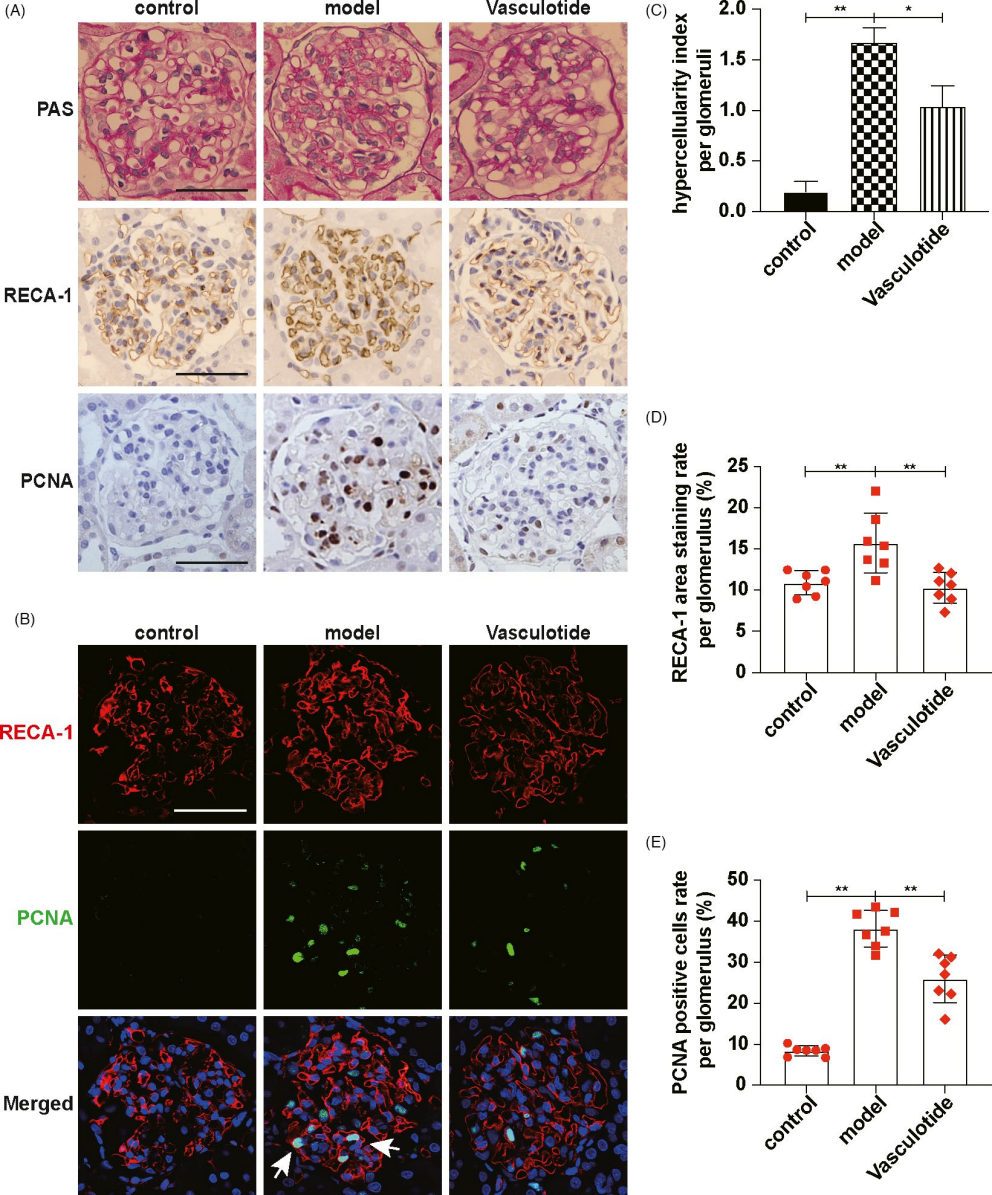
Enhancing Tie2 phosphorylation can effectively alleviate ECs proliferation in anti‐Thy‐1 nephritis. A, PAS staining showed cell number per glomerulus. RECA‐1 and PCNA expressions in glomeruli were detected by immunohistochemical staining. B, Locations of RECA‐1 and PCNA expressions were showed by immunofluorescence co‐staining. White arrows indicated proliferating ECs. RECA‐1, red. PCNA, green. DAPI, blue. C, Hypercellularity index per glomerulus was evaluated (n = 7). D, RECA‐1 area staining rate per glomerulus was qualified (n = 7). E, PCNA‐positive cells rate per glomerulus was qualified (n = 7). Results are presented as the mean values (±SD), *P < .05, **P < .01. Scale bars, 50 μm. ECs, endothelial cells; PCNA, proliferative cells nuclear antigen; RECA‐1, rat endothelial cells antigen‐1; Tie2, TEK tyrosine kinase. Control, normal rats. Model, anti‐Thy‐1 nephritis rats. Vasculotide, anti‐Thy‐1 nephritis rats treated by Vasculotide

In vitro experiments, we verified that MCs promoted ECs proliferation through VEGFA/VEGFR2 and Angpt2/Tie2 pathway and the advance of Tie2 phosphorylation could control ECs proliferation. To further explore whether increasing Tie2 phosphorylation can effectively alleviate ECs proliferation in vivo, we established the anti‐Thy‐1 nephritis and administered Vasculotide (enhancing Tie2 phosphorylation). The rats were divided into the control group, model group and Vasculotide group (Figure S6A). First of all, Western blot results indicated that Vasculotide can increase Tie2 phosphorylation (Figure S6B). PAS staining results showed that the hypercellularity index per glomerulus decreased significantly after Vasculotide treatment (Figure 6A,C). RECA‐1 and PCNA immunohistochemical staining (Figure 6A,D,E) and immunofluorescence co‐staining (Figure 6B) results showed that ECs proliferation was alleviated by Vasculotide. Western blot results showed that after Vasculotide treatment, the expression of CD34 was decreased (Figure S6C), which indicated that Vasculotide could decrease ECs proliferation. Thus, by summarizing experiment results, we concluded that promoting Tie2 phosphorylation by Vasculotide could effectively alleviate ECs proliferation in anti‐Thy‐1 nephritis.

## 4 DISCUSSION

Mesangial proliferative glomerulonephritis (MPGN) does not represent a specific glomerulopathy. As a pathological description of a class of glomerular diseases with mesangial hyperplasia, it can occur in primary glomerulonephritis (IgA nephropathy, lupus nephritis, infectious glomerulonephritis, IgM nephropathy, etc) and systemic disease (kidney allograft, diabetes, immune complex deposition, etc).^27^, ^28^ MPGN is one of the major factors of chronic kidney disease which leads to end‐stage renal disease. Therefore, the study on its mechanism can provide new ideas for the treatment of a class of kidney diseases. Studies related to experimental MPGN have shown that when mesangial cells (MCs) damage and mesangial matrix accumulate, endothelial cells (ECs) proliferate increasingly.^29^, ^30^ This indicates that there might be cell‐cell communication between MCs and ECs in experimental MPGN. To investigate the specific mechanism involved in the cell‐cell communication, we established a co‐culture system in vitro, which showed that PDGF‐BB‐activated MCs promoted ECs proliferation. So, we wondered if we could alleviate disease progression by blocking such communication.

Imbalanced vascular endothelial growth factor (VEGF) expression in glomeruli has been demonstrated in a variety of primary and acquired renal diseases.^31^ VEGFA plays a key role in maintaining the glomerular filtration barrier by affecting apoptosis, proliferation and differentiation of ECs.^32^, ^33^, ^34^ And VEGFA is also involved in the inflammatory cascade by increasing vascular permeability, leukocyte adhesion, and intercellular cell adhesion molecule1 (ICAM‐1) and vascular cell adhesion molecule1 (VCAM‐1) expression.^35^ In diabetic nephropathy, VEGFA is involved in VEGF/nephrin pathway, VEGF/nitric oxide synthase pathway, insulin resistance and angiopoietin expression.^36^ Urinary VEGFA levels are significantly higher in lupus nephritis patients than in lupus patients without renal damage.^37^ Our study also found that VEGFA expression increased along with ECs proliferation on the 7th day of anti‐Thy‐1 nephritis. For anti‐Thy‐1 nephritis is dominated by mesangial lesions, and it has been reported that activated MCs could produce VEGFA^38^ which was verified by co‐staining VEGFA with the protein marker of MCs (Thy‐1) or the protein marker of podocytes (WT‐1, Wilms tumour protein1) in glomeruli. Our results indicated that VEGFA expression increased significantly in the mesangial area of anti‐Thy‐1 nephritis. Therefore, we hypothesized that ECs proliferation was related to the expression of VEGFA in MCs. To further elucidate whether VEGFA is involved in signal transduction between MCs and ECs, VEGFA neutralizing antibody was added to the co‐culture system. The results showed that VEGFA and its receptor VEGFR2 induced cell‐cell communication between MCs and ECs.

Angiopoietins belong to a family of growth factors and include Angpt1, Angpt2 and Angpt3. Angpt2 plays an important role in kidney disease, because it was increased in serum of patients with chronic kidney disease, lupus nephritis, thrombotic microangiopathy or anti‐glomerular basement membrane nephropathy,^39^, ^40^, ^41^ and associated with poor prognosis in hypertension and coronary heart disease and also associated with high risk of acute kidney injury in critically ill patients.^42^ The expression of Angpt2 of fibrotic kidneys increased, which stimulated the production of chemokines and adhesion factors in aortic, increased the infiltration of Ly6C(Low) macrophages and promoted the expression of fibrotic cytokine TGF‐β1.^35^ Studies on diabetic nephropathy indicated that Angpt2 promotes autophagy in MCs through miR‐33‐5p/SOCS5 loop.^36^ In our study, we found that Angpt2 expression increased on the 7th day of anti‐Thy‐1 nephritis. And by co‐staining Angpt2 with protein marker of ECs (RECA‐1, rat endothelial cells antigen‐1) or protein marker of MCs (Thy‐1), we verified that Angpt2 was expressed by ECs. Relevant studies have shown that Angpt2 collaborates with VEGFA to promote angiogenesis.^43^, ^44^ In breast cancer, non‐small cell lung cancer and acute myeloid leukaemia, the expression of VEGFA and Angpt2 increased simultaneously, which promoted tumour growth. High VEGFA expression is considered as the prerequisite for Angpt2‐promoted hepatoma carcinoma cells growth.^45^ This study confirmed that VEGFA expressed in MCs promoted Angpt2 expression in ECs which promoted ECs proliferation. However, in developing retinas and ischaemic retinal models, it was demonstrated that Angpt2 promotes the effect of VEGFA on retinal angiogenesis.^38^ Thus, although VEGFA and Angpt2 have synergistic effects in promoting angiogenesis, the relationship between them is controversial in different organs.

Tie2 (TEK Receptor Tyrosine Kinase) expresses on ECs surface. Angpt1 and Angpt2, as two ligands of Tie2, affect ECs survival, proliferation, migration and inflammation pathways by influencing Tie2 activation.^46^ Current studies have shown that Tie2 can act as a switch to regulate vascular leakage and organ inflammation, but the role of Tie2 activation in different diseases remains highly controversial.^47^, ^48^ Therefore, we summarized 20 studies on the effect of Tie2 activation on angiogenesis in different diseases in the past 5 years and attempted to have a more comprehensive understanding of Tie2 (Figure S8). By summarizing the literatures, we found that the studies on Tie2 activation and angiogenesis are focused on brain, tumour, sepsis and ophthalmic diseases. Among them, most studies have shown that Tie2 activation can reduce permeability and inflammation, stabilize blood vessels and maintain the vascular function (such as stabilizing the blood‐brain barrier,^49^, ^50^ reducing inflammation and dysregulation of coagulation in sepsis,^51^ inhibiting the metastasis of tumour regeneration,^52^ and repairing Schlemm's canal integrity and reducing intraocular pressure^53^, ^54^). However, three studies have suggested that Tie2 activation can promote angiogenesis, two of which mentioned that Tie2 activation promoted vascular repair in diabetes^55^ and promoted choroidal angiogenesis to relieve hypoxia.^56^ And one mentioned that Tie2 activation promoted angiogenesis to increase treatment resistance of tumour, which could be seen as “tumour self‐preservation behavior.” Therefore, it is obvious that Tie2 plays different roles in different diseases. We need to intervene Tie2 activation based on the pathological changes of diseases to achieve the effect of alleviating diseases. In this study, we added Angpt1 or Angpt2 to the co‐culture system of MCs and ECs in vitro. The results showed that with the decline of Tie2 activation after adding Angpt2, the proliferation ability of ECs increased. On the contrary, Tie2 phosphorylation increased after adding Angpt1, but the proliferation ability of ECs decreased. In conclusion, Tie2 phosphorylation is negatively correlated with ECs proliferation.

The results showed that VEGFA/VEGFR2 and Angpt2/Tie2 participated in the cell‐cell communication between MCs and ECs. Therefore, we can inhibit ECs proliferation by blocking VEGFA or promoting Tie2 phosphorylation. As early as 1999, Tammo Ostendorf et al applied VEGFA‐antagonist to anti‐Thy‐1 nephritis, which though effectively inhibited ECs proliferation, inhibited ECs restoration and increased ECs death at the same time.^38^ It seems that alleviating the pathological changes of the glomerulus by blocking VEGFA is not advisable. So, we attempted to inhibit ECs proliferation by promoting Tie2 phosphorylation. A recently developed drug, Vasculotide, enhancing Tie2 phosphorylation, has been applied to a variety of diseases.^24^, ^57^, ^58^ It was reported that Vasculotide could alleviate haemorrhagic shock, tumour cells metastasis and acute kidney injury by protecting ECs.^24^, ^26^, ^59^ So, we applied Vasculotide to anti‐Thy‐1 nephritis. And the results showed that the phosphorylation of Tie2 was promoted and ECs proliferation was significantly inhibited, which suggests a possible clinical application for Vasculotide in MPGN.

In summary, the present study found that the VEGFA/VEGFR2 and Angpt2/Tie2 signalling pathway was involved in the cell‐cell communication between MCs and ECs. Moreover, in vivo experiments showed that enhancing Tie2 phosphorylation by Vasculotide could alleviate ECs proliferation on the 7th day of anti‐Thy‐1 nephritis, which provided a new idea and strategy for alleviating the vascular lesions in MPGN.

## CONFLICT OF INTEREST

All of the authors declared no competing interests.

## AUTHORS' CONTRIBUTIONS

Yinghua Zhao, Lingling Wu and Xiangmei Chen designed research; Yinghua Zhao, Bo Fu and Pu Chen analysed data and performed research; Yinghua Zhao and Lingling Wu wrote the paper; Qinggang Li, Qing Ouyang, Chuyue Zhang, Guangyan Cai and Xiangmei Chen optimized the paper.

## Supporting information

Figure S1

Figure S2

Figure S3

Figure S4

Figure S5

Figure S6

Figure S7

Figure S8

Supplementary Material

Supplementary Material

## Data Availability

All data and models generated and used during the study are available from the corresponding author by request.
