# Supplementary figures and images for: Activated mesangial cells induce glomerular endothelial cells proliferation in rat anti‐Thy‐1 nephritis through VEGFA/VEGFR2 and Angpt2/Tie2 pathway
Source: Cell Prolif. 2021 May 13;54(6):e13055. doi: 10.1111/cpr.13055 (PMC8168418; doi:10.1111/cpr.13055)

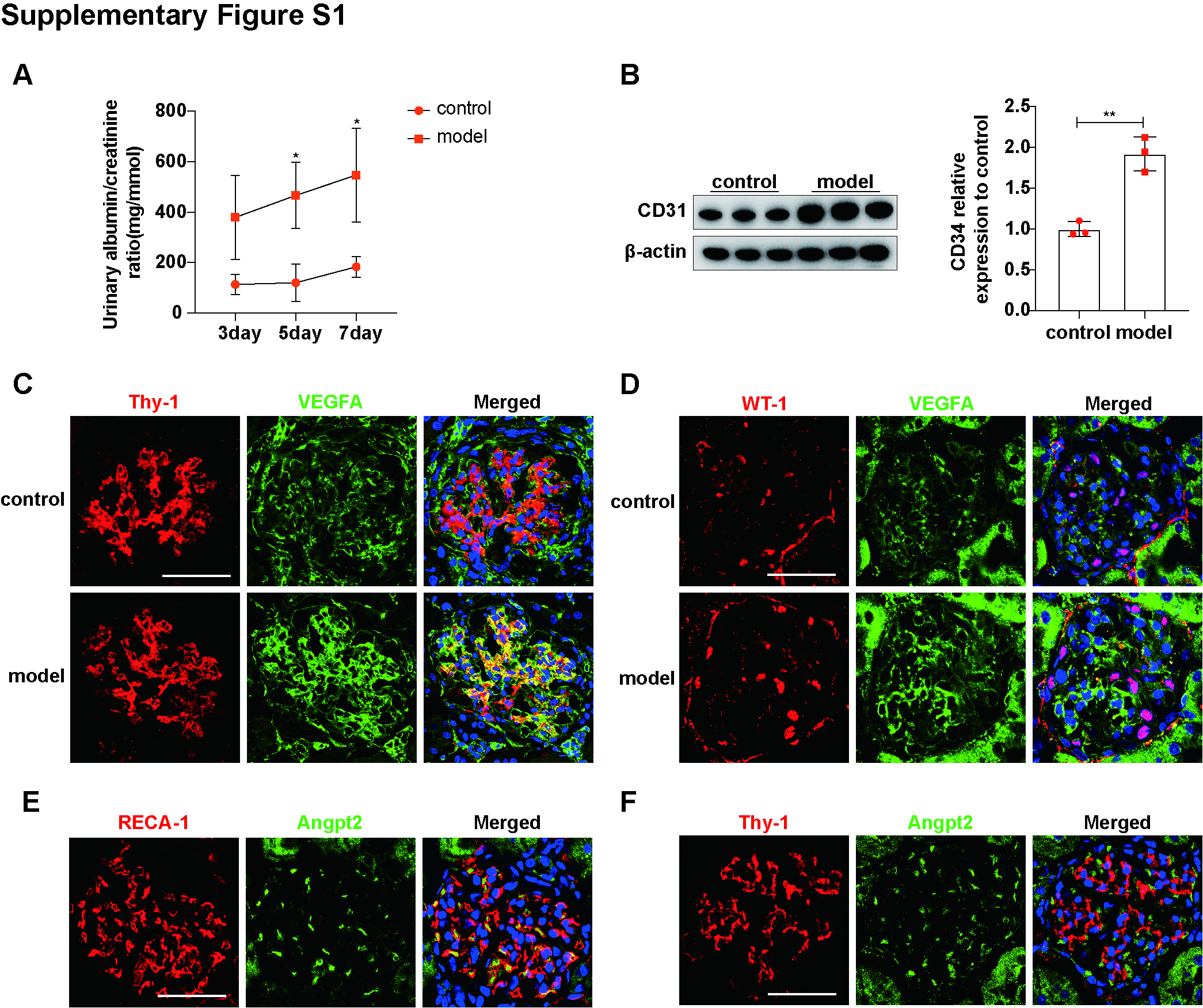

Supplement: Supplementary file 1 — Figure S1 [file CPR-54-e13055-s004.tif]

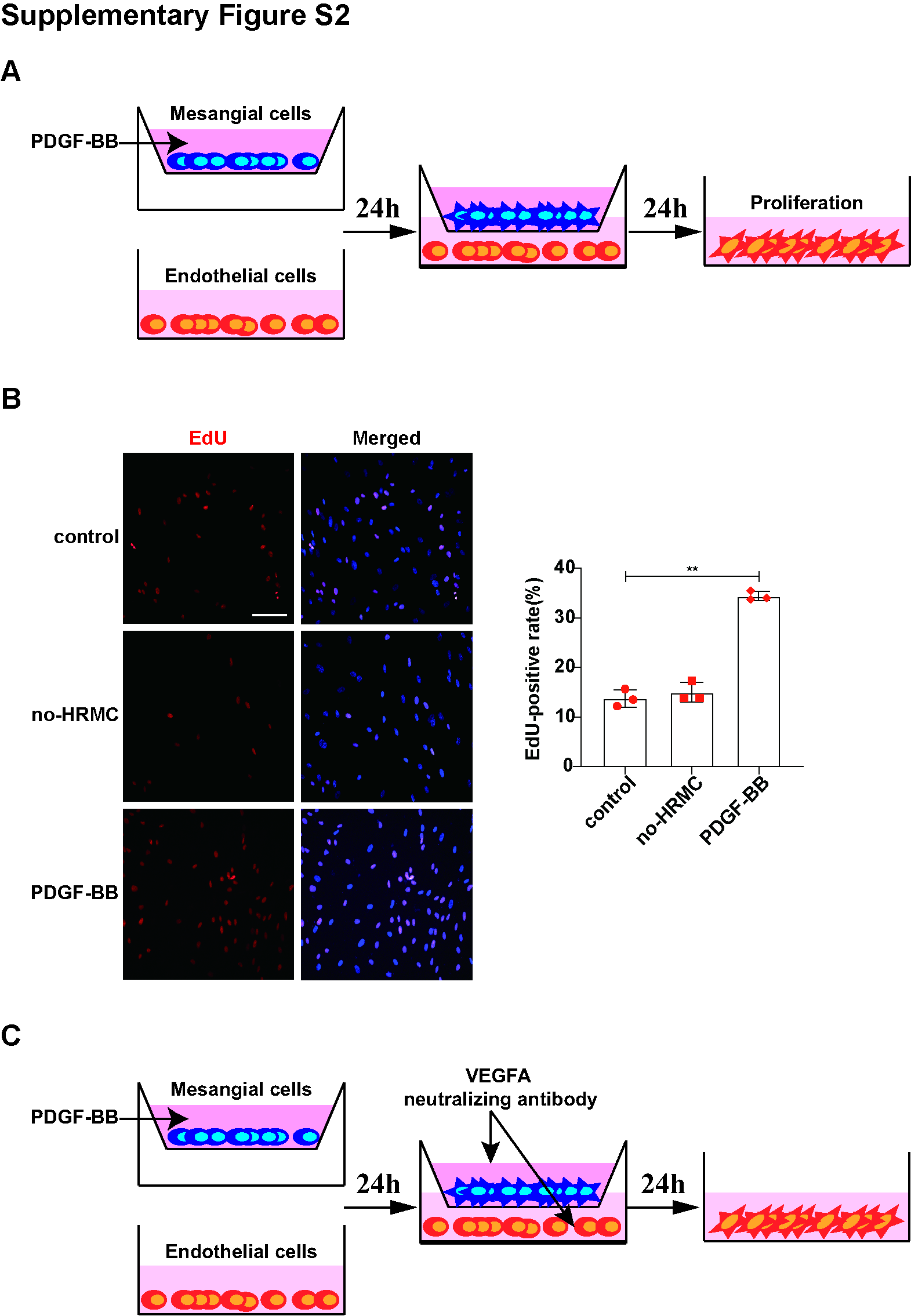

Supplement: Supplementary file 2 — Figure S2 [file CPR-54-e13055-s005.tif]

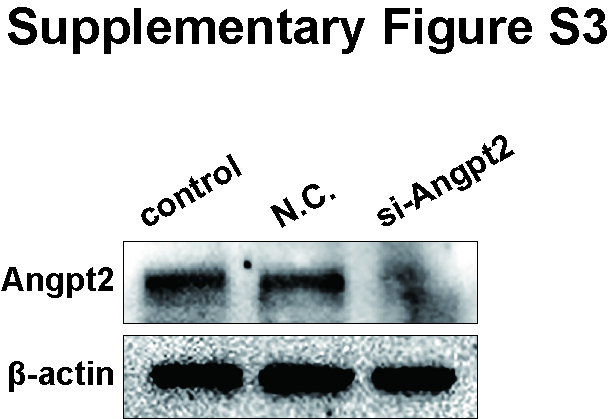

Supplement: Supplementary file 3 — Figure S3 [file CPR-54-e13055-s008.tif]

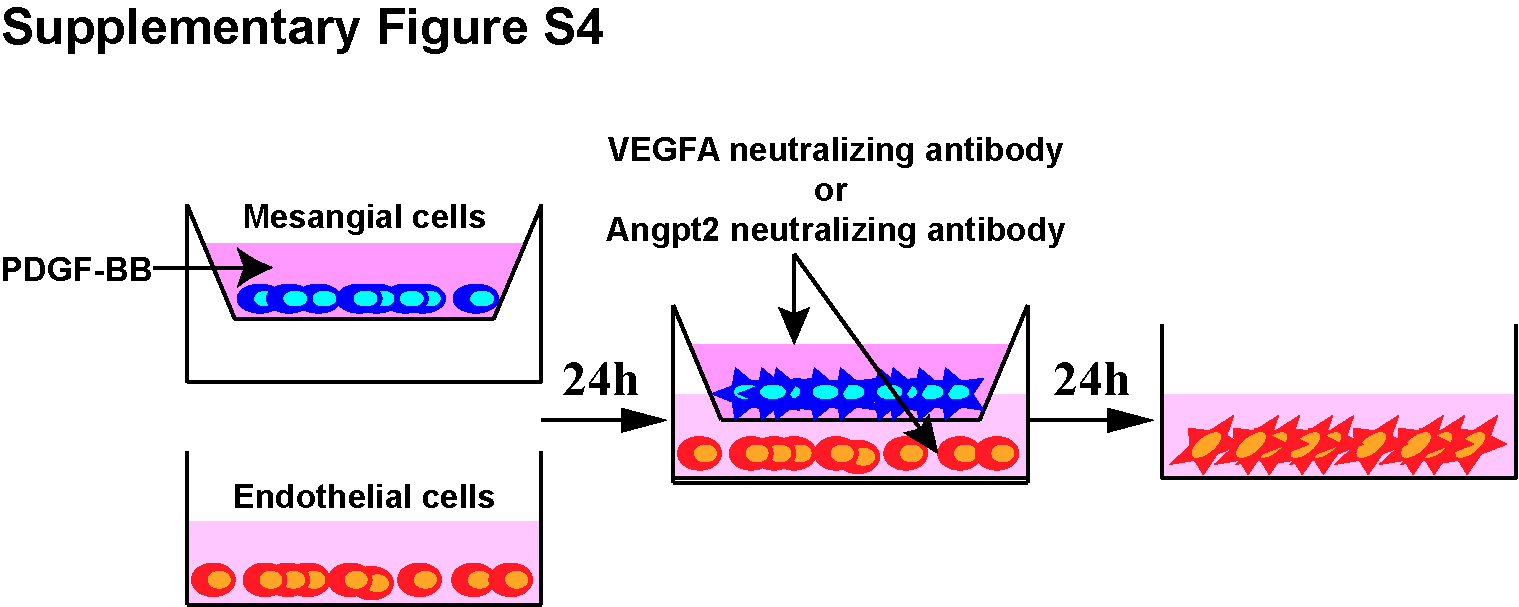

Supplement: Supplementary file 4 — Figure S4 [file CPR-54-e13055-s006.tif]

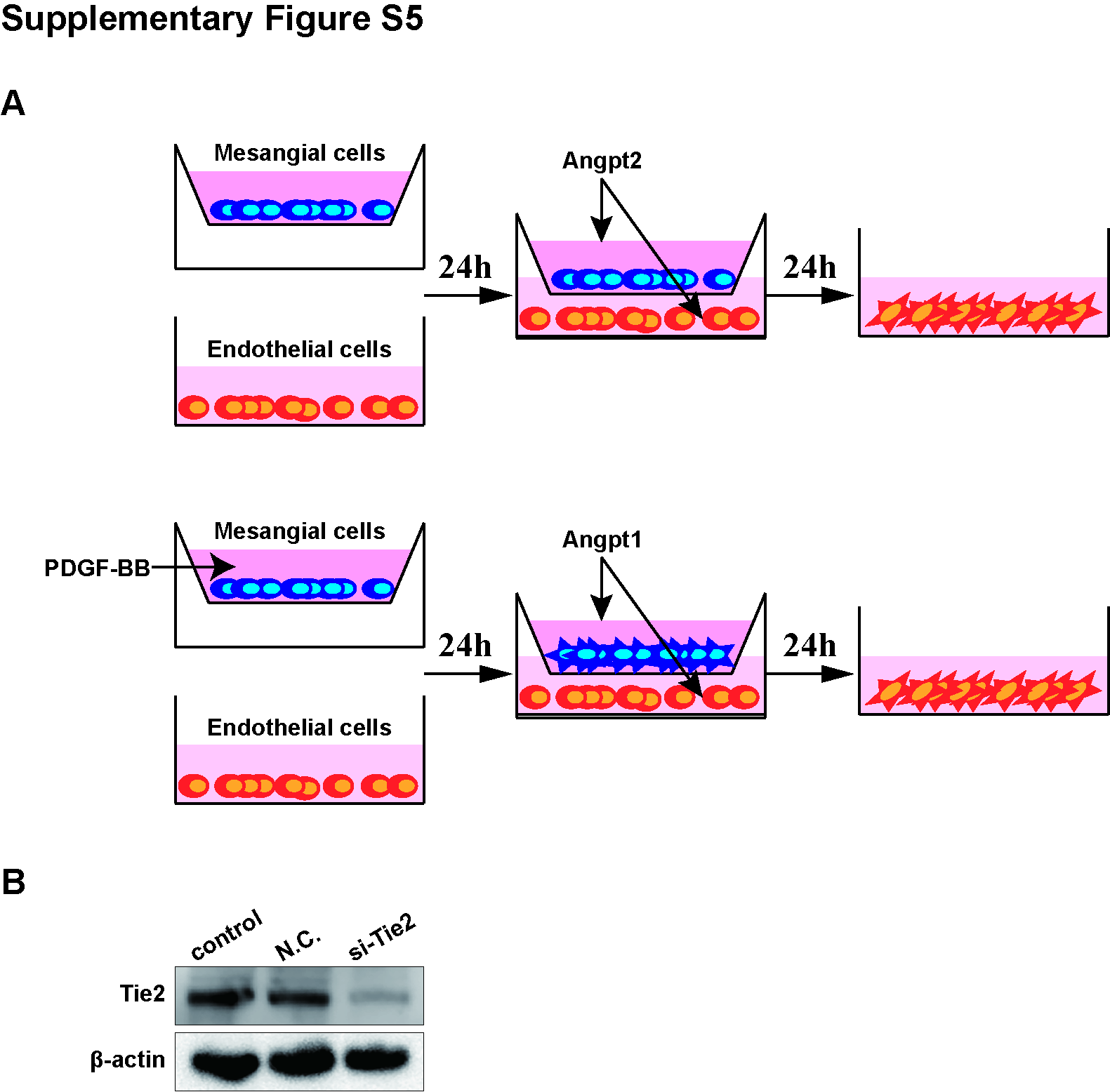

Supplement: Supplementary file 5 — Figure S5 [file CPR-54-e13055-s001.tif]

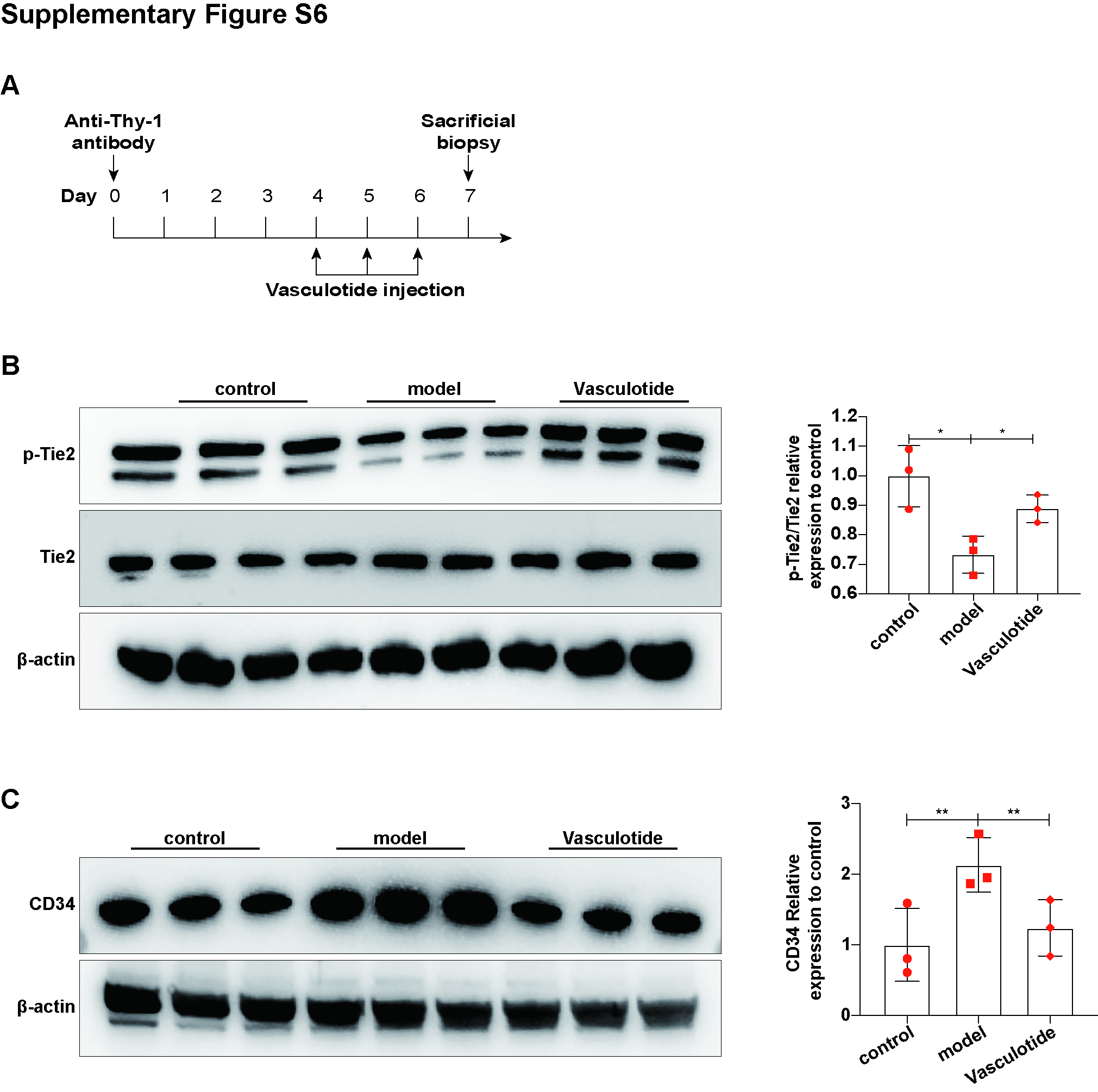

Supplement: Supplementary file 6 — Figure S6 [file CPR-54-e13055-s010.tif]

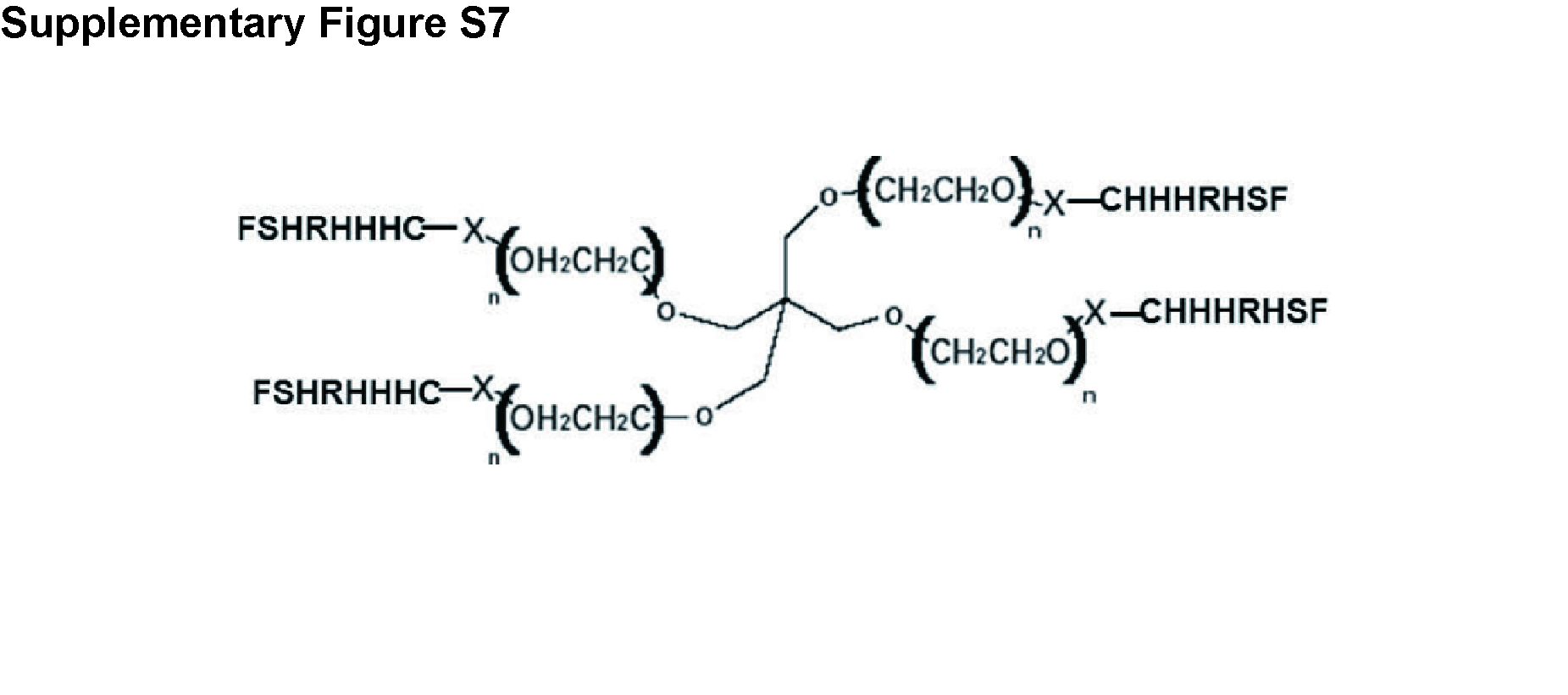

Supplement: Supplementary file 7 — Figure S7 [file CPR-54-e13055-s007.tif]

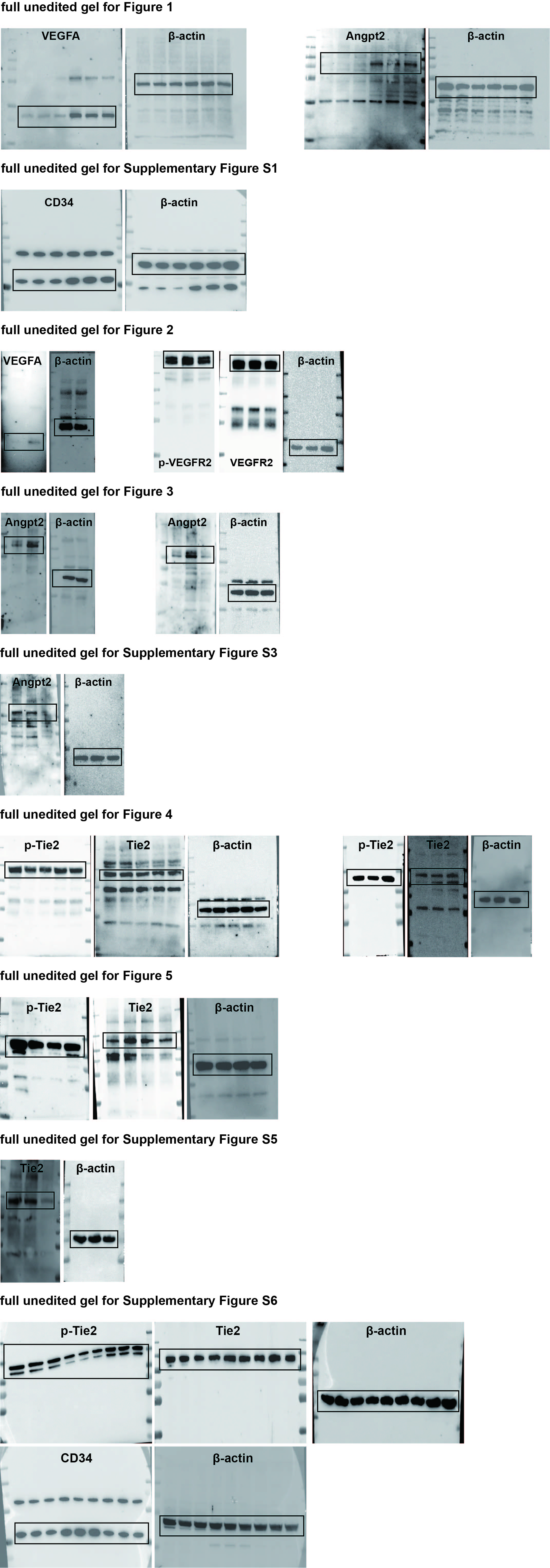

Supplement: Supplementary file 9 — Supplementary Material [file CPR-54-e13055-s003.jpg]
