# Supplementary material for: Activated mesangial cells induce glomerular endothelial cells proliferation in rat anti‐Thy‐1 nephritis through VEGFA/VEGFR2 and Angpt2/Tie2 pathway
Source: Cell Prolif. 2021 May 13;54(6):e13055. doi: 10.1111/cpr.13055 (PMC8168418; doi:10.1111/cpr.13055)
Supplement: Supplementary file 8 — Figure S8 [file CPR-54-e13055-s009.docx]

| **Research Fields** | **Research Contents** | **Roles of Tie2 activation  on blood vessels** | **Reference** |
| --- | --- | --- | --- |
| Brain | Angiopoietin‑2‑induced blood–brain barrier compromise and increased stroke size are rescued by VE‑PTP‑dependent restoration of Tie2 signaling | Reduce permeability and maintain the vascular function | Stefanie Gurnik, et al. 2016[1] |
|  | Angiopoietin/Tie2 axis regulates the age-at-Injury cerebrovascular response to traumatic brain injury | Maintain blood-brain barrier stability | Thomas R. Brickler, et al. 2018[2] |
| Diabetes | Targeting VE-PTP phosphatase protects the kidney from diabetic injury | Reduce permeability and inflammation | Isabel A. Carota, et al. 2019[3] |
|  | Improved angiogenic activity of endothelial progenitor cell in diabetic patients treated with insulin plus metformin | Improve angiogenic activity of endothelial progenitor cell | Simin Asadian, et al. 2018[4] |
| Eyes | Impaired angiopoietin/Tie2 signaling compromises Schlemm’s canal integrity and induces glaucoma | Repair schlemm’s canal integrity and transcytosis, reduced intraocular pressure | Jaeryung Kim, et al. 2017[5] |
|  | A Small Molecule Inhibitor of VE-PTP Activates Tie2 in Schlemm’s Canal Increasing Outflow Facility and Reducing Intraocular Pressure | Repair schlemm’s canal integrity and transcytosis and reduced intraocular pressure. | Jaeryung Kim, et al. 2019[6] |
|  | Tie2 activation promotes choriocapillary regeneration for alleviating neovascular age-related macular degeneration | Suppress choroidal neovascularization and vascular leakage but also regenerate the choriocapillaris and relieved hypoxia. | Guorong Li, et al. 2020[7] |
| **Research Fields** | **Research Contents** | **Roles of Tie2 activation  on blood vessels** | **Reference** |
| Tumor | Tie2-mediated vascular remodeling by ferritin-based protein C nanoparticles confers antitumor and anti-metastatic activities | stabilize blood vessels and inhibit tumor growth | Young Sun Choi, et al. 2020[8] |
|  | Targeting the ERβ/Angiopoietin-2/Tie-2 signaling mediated angiogenesis with the FDA-approved anti-estrogen Faslodex to increase the Sunitinib sensitivity in RCC | promote the angiogenesis and increase treatment resistance | Junfei Gu, et al. 2020[9] |
|  | Pericyte-expressed Tie2 controls angiogenesis and vessel maturation | limit endothelial sprouting, tumor growth and tumor angiogenesis. | Martin Teichert, et al. 2017[10] |
|  | Normalization of tumor vessels by Tie2 activation and ang2 inhibition enhances drug delivery and produces a favorable tumor microenvironment | enhance blood perfusion and reduce tumor growth and metastasis. | Jin-Sung Park, et al. 2016[11] |
|  | 3‐O‐Acetyloleanolic acid inhibits angiopoietin‐1‐induced angiogenesis and lymphangiogenesis via suppression of angiopoietin‐1/Tie‐2 signaling | induce tumor growth, angiogenesis, and lymphangiogenesis | Jeon Hwang‐Bo, et al. 2019[12] |
|  | Novel Tie2 inhibitor Bbay-826 displays in vivo efficacy in experimental syngeneic murine glioma models | is harmful for the treatments of highly vascular tumors | Hannah schneider, et al. 2017[13] |
| **Research Fields** | **Research Contents** | **Roles of Tie2 activation  on blood vessels** | **Reference** |
| Sepsis | Identification of specific Tie2 cleavage sites and therapeutic modulation in experimental sepsis | reduce vascular permeability | Temitayo O Idowu, et al. 2020[14] |
|  | Tie2 protects the vasculature against thrombus formation in systemic inflammation | normalize coagulation in inflammatory states while averting the bleeding risks | Sarah J. Higgins, et al. 2018[15] |
|  | Tie2 Activation Promotes Protection and Reconstitution of the Endothelial Glycocalyx in Human Sepsis | reduce vascular leakage | Carolin Christina Drost, et al. 2019[16] |
|  | Molecular regulation of acute Tie2 suppression in sepsis | promote vascular homeostasis during quiescence | Kristina Thamm, et al. 2019[17] |
| Ischemic disease | A collagen IV–derived peptide disrupts α5β1 integrin and potentiates Ang2/Tie2 signaling | inhibit vascular leakage | Adam C. Mirando, et al. 2019[18] |
|  | Loss of Endothelial Glycocalyx Hyaluronan Impairs Endothelial Stability and Adaptive Vascular Remodeling after Arterial Ischemia | inhibit excessive angiogenesis. | Gangqi Wang, et al. 2020[19] |
| Scrub Typhus | Polarized lung inflammation and Tie2/angiopoietin-mediated endothelial dysfunction during severe Orientia tsutsugamushi infection | reduce vascular activation and tissue damage. | Brandon Trent, et al. 2020[20] |

**Reference**

1. Gurnik, S., et al., Angiopoietin-2-induced blood-brain barrier compromise and increased stroke size are rescued by VE-PTP-dependent restoration of Tie2 signaling. Acta neuropathologica, 2016. 131(5): p. 753-773.

2. Brickler, T.R., et al., Angiopoietin/Tie2 Axis Regulates the Age-at-Injury Cerebrovascular Response to Traumatic Brain Injury. The Journal of neuroscience : the official journal of the Society for Neuroscience, 2018. 38(45): p. 9618-9634.

3. Carota, I.A., et al., Targeting VE-PTP phosphatase protects the kidney from diabetic injury. The Journal of experimental medicine, 2019. 216(4): p. 936-949.

4. Asadian, S., et al., Improved angiogenic activity of endothelial progenitor cell in diabetic patients treated with insulin plus metformin. Journal of cellular biochemistry, 2018.

5. Kim, J., et al., Impaired angiopoietin/Tie2 signaling compromises Schlemm's canal integrity and induces glaucoma. The Journal of clinical investigation, 2017. 127(10): p. 3877-3896.

6. Li, G., et al., A Small Molecule Inhibitor of VE-PTP Activates Tie2 in Schlemm's Canal Increasing Outflow Facility and Reducing Intraocular Pressure. Investigative ophthalmology & visual science, 2020. 61(14): p. 12.

7. Kim, J., et al., Tie2 activation promotes choriocapillary regeneration for alleviating neovascular age-related macular degeneration. Science advances, 2019. 5(2): p. eaau6732.

8. Choi, Y.S., et al., Tie2-mediated vascular remodeling by ferritin-based protein C nanoparticles confers antitumor and anti-metastatic activities. Journal of hematology & oncology, 2020. 13(1): p. 123.

9. Gu, J., et al., Targeting the ERβ/Angiopoietin-2/Tie-2 signaling-mediated angiogenesis with the FDA-approved anti-estrogen Faslodex to increase the Sunitinib sensitivity in RCC. Cell death & disease, 2020. 11(5): p. 367.

10. Teichert, M., et al., Pericyte-expressed Tie2 controls angiogenesis and vessel maturation. Nature communications, 2017. 8: p. 16106.

11. Park, J.-S., et al., Normalization of Tumor Vessels by Tie2 Activation and Ang2 Inhibition Enhances Drug Delivery and Produces a Favorable Tumor Microenvironment. Cancer cell, 2016. 30(6): p. 953-967.

12. Hwang-Bo, J., J.-H. Park, and I.S. Chung, 3-O-Acetyloleanolic acid inhibits angiopoietin-1-induced angiogenesis and lymphangiogenesis via suppression of angiopoietin-1/Tie-2 signaling. Phytotherapy research : PTR, 2020. 34(2): p. 359-367.

13. Schneider, H., et al., Novel TIE-2 inhibitor BAY-826 displays in vivo efficacy in experimental syngeneic murine glioma models. Journal of neurochemistry, 2017. 140(1): p. 170-182.

14. Idowu, T.O., et al., Identification of specific Tie2 cleavage sites and therapeutic modulation in experimental sepsis. eLife, 2020. 9.

15. Higgins, S.J., et al., Tie2 protects the vasculature against thrombus formation in systemic inflammation. The Journal of clinical investigation, 2018. 128(4): p. 1471-1484.

16. Drost, C.C., et al., Tie2 Activation Promotes Protection and Reconstitution of the Endothelial Glycocalyx in Human Sepsis. Thrombosis and haemostasis, 2019. 119(11): p. 1827-1838.

17. Thamm, K., et al., Molecular Regulation of Acute Tie2 Suppression in Sepsis. Critical care medicine, 2018. 46(9): p. e928-e936.

18. Mirando, A.C., et al., A collagen IV-derived peptide disrupts α5β1 integrin and potentiates Ang2/Tie2 signaling. JCI insight, 2019. 4(4).

19. Wang, G., et al., Loss of Endothelial Glycocalyx Hyaluronan Impairs Endothelial Stability and Adaptive Vascular Remodeling After Arterial Ischemia. Cells, 2020. 9(4).

20. Trent, B., et al., Polarized lung inflammation and Tie2/angiopoietin-mediated endothelial dysfunction during severe Orientia tsutsugamushi infection. PLoS neglected tropical diseases, 2020. 14(3): p. e0007675.
