# Supplementary material for: Activated mesangial cells induce glomerular endothelial cells proliferation in rat anti‐Thy‐1 nephritis through VEGFA/VEGFR2 and Angpt2/Tie2 pathway
Source: Cell Prolif. 2021 May 13;54(6):e13055. doi: 10.1111/cpr.13055 (PMC8168418; doi:10.1111/cpr.13055)
Supplement: Supplementary file 10 — Supplementary Material [file CPR-54-e13055-s002.docx]

**Supplementary Figure S1.** A, Qualifications of urine albumin/creatinine ratio (UACR). B, CD34 expressions in glomeruli were detected by Western blot. (C, D) Locations of VEGFA expression in glomeruli were detected by Thy-1/VEGFA or WT-1/VEGFA co-staining immunofluorescence. (E, F) Locations of Angpt2 expression in glomeruli were detected by RECA-1/Angpt2 or Thy-1/Angpt2 co-staining immunofluorescence. Results are presented as the mean values (±SD), *P<0.05, **P<0.01. Scale bars, 50um. CD34, protein marker of ECs. VEGFA, vascular endothelial growth factor A. MCs, mesangial cells. Angpt2, angiopoietin2. ECs, endothelial cells. RECA-1, rat endothelial cells antigen-1. WT-1, Wilms tumor protein 1, protein marker of podocyte. control, normal rats. model, anti-Thy-1 nephritis rats.

**Supplementary Figure S2.** A, MCs were seeded in transwell inserts and were cultured alone or stimulated with PDGF-BB treatment for 24h and were then washed with fresh media followed by co-culturing with ECs for another 24h. B, EdU was used to detect ECs proliferation. C, VEGFA neutralizing antibody was added in co-culture system. Results are presented as the mean values (±SD), **P<0.01. Scale bar, 50um. MCs, mesangial cells. HRMCs, human renal mesangial cells. HRGECs, human renal glomerular endothelial cells. PDGF-BB, platelet derived growth factor BB, to activate MCs. VEGFA, vascular endothelial growth factor A. control, ECs co-cultured with inactive MCs. no-HRMC, ECs cultured alone. PDGF-BB, ECs co-cultured with PDGF-BB-activated MCs.

**Supplementary Figure S3.** Angpt2 expressions in ECs were detected by Western blot. control, normal ECs. N.C., ECs transfected with negative control siRNA. si-Angpt2, ECs transfected with si-Angpt2. Angpt2, angiopoietin2. ECs, endothelial cells.

**Supplementary Figure S4.** MCs were seeded in transwell inserts and were cultured alone or stimulated with PDGF-BB treatment for 24h and were then washed with fresh media followed by co-culturing with ECs for another 24h. IgG antibody, Angpt2 neutralizing antibody or VEGFA neutralizing antibody was added in co-culture system. HRMCs, human renal mesangial cells. HRGECs, human renal glomerular endothelial cells. PDGF-BB, platelet derived growth factor BB, to activate MCs. Angpt2, angiopoietin2.

**Supplementary Figure S5.** A, MCs were seeded in transwell inserts and were cultured alone or stimulated with PDGF-BB treatment for 24h and were then washed with fresh media followed by co-culturing with ECs for another 24h. Recombinant human Angpt1 or Angpt2 was added in co-culture system. B, Tie2 expressions on ECs were detected by Western blot. control, normal ECs. N.C., ECs transfected with negative control siRNA. si-Tie2, ECs transfected with si-Tie2. HRMCs, human renal mesangial cells. HRGECs, human renal glomerular endothelial cells. PDGF-BB, platelet derived growth factor BB, to activate MCs. Angpt1, angiopoietin 1. Angpt2, angiopoietin 2. Tie2, TEK tyrosine kinase.

**Supplementary Figure S6.** A, After anti-Thy-1 antibody injection, Vasculotide was injected continuously on the 4^th^, 5^th^ and 6^th^ days, and the rats were sacrificed on the 7^th^ day. B, p-Tie2 and Tie2 expressions were detected by Western blot. C, CD34 expression were detected by Western blot. Results are presented as the mean values (±SD), *<0.05, **P<0.01. Tie2, TEK tyrosine kinase. CD34, protein marker of ECs. control, normal rats. model, anti-Thy-1 nephritis rats.

**Supplementary Figure S7.** Schematic structure of Vasculotide.

**Supplementary S8.** The summarizing of the relationship between Tie2 activation and angiogenesis.
